# 

**Published:** 1928

**Authors:** 


### CONTENTS.

| | Page |
|---|---|
| **Some Developments in Dermatology during the last Thirty Years.** By W. Kenneth Wills, O.B.E., M.B., B.Ch. .. .. | 1 |
| **The Long Fox Memorial Lecture: The Reality of Delusions.** By Henry Devine, O.B.E., M.D., B.S., F.R.C.P. .. .. .. | 19 |
| **Problems in the Treatment of Hæmoptysis.** By Hugh H. Carleton, M.A., M.D., M.R.C.P. .. .. .. .. .. .. | 39 |
| **Recent Experiences of Intussusception.** By Hubert Chitty, M.S., F.R.C.S. .. .. .. .. .. .. .. .. | 51 |
| **Reviews of Books** .. .. .. .. .. .. .. .. | 59 |
| **Editorial Notes** .. .. .. .. .. .. .. .. | 68 |
| **Meetings of Societies** .. .. .. .. .. .. .. | 71 |
| **The Medical Library of the University of Bristol** .. .. | 75 |
| **Local Medical Notes** .. .. .. .. .. .. .. .. | 76 |

